# Autophagy Hijacking in PBMC From COVID-19 Patients Results in Lymphopenia

**DOI:** 10.3389/fimmu.2022.903498

**Published:** 2022-05-30

**Authors:** Cristiana Barbati, Alessandra Ida Celia, Tania Colasanti, Marta Vomero, Mariangela Speziali, Erisa Putro, Giorgia Buoncuore, Flavia Savino, Serena Colafrancesco, Federica Maria Ucci, Claudia Ciancarella, Eugenia Balbinot, Susanna Scarpa, Francesco Natalucci, Greta Pellegrino, Fulvia Ceccarelli, Francesca Romana Spinelli, Claudio Maria Mastroianni, Fabrizio Conti, Cristiano Alessandri

**Affiliations:** ^1^ Arthritis Center, Dipartimento di Scienze Cliniche Internistiche, Anestesiologiche e Cardiovascolari, Sapienza University of Rome, Rome, Italy; ^2^ Rheumatology, Immunology and Clinical Medicine Unit, Department of Medicine, Università Campus Bio-Medico di Roma, Rome, Italy; ^3^ Department of Experimental Medicine, Sapienza University, Rome, Italy; ^4^ Department of Public Health and Infectious Diseases, Sapienza, University of Rome, Rome, Italy

**Keywords:** autophagy, apoptosis, inflammation, lymphocytes, SARS-CoV-2, COVID-19

## Abstract

Autophagy is a homeostatic process responsible for the self-digestion of intracellular components and antimicrobial defense by inducing the degradation of pathogens into autophagolysosomes. Recent findings suggest an involvement of this process in severe acute respiratory syndrome coronavirus 2 (SARS-CoV-2) infection. However, the role of autophagy in the immunological mechanisms of coronavirus disease 2019 (COVID-19) pathogenesis remains largely unexplored. This study reveals the presence of autophagy defects in peripheral immune cells from COVID-19 patients. The impairment of the autophagy process resulted in a higher percentage of lymphocytes undergoing apoptosis in COVID-19 patients. Moreover, the inverse correlation between autophagy markers levels and peripheral lymphocyte counts in COVID-19 patients confirms how a defect in autophagy might contribute to lymphopenia, causing a reduction in the activation of viral defense. These results provided intriguing data that could help in understanding the cellular underlying mechanisms in COVID-19 infection, especially in severe forms.

## Introduction

Severe acute respiratory syndrome coronavirus 2 (SARS-CoV-2) is a highly transmissible coronavirus that emerged in late 2019 and caused a pandemic of acute respiratory disease, named “coronavirus disease 2019” (COVID-19), which still now threatens human health and public safety (1).

In severe COVID-19 patients, both adaptive and innate immune responses that are critical for antiviral reactions were described as impaired (2).

In particular, lymphopenia (lymphocyte count <1.0 × 10^9^/L)^3^ and inflammatory cytokine storm are typical abnormalities found in COVID-19 patients, probably associated with disease severity, highlighting the strong SARS-CoV-2 ability in suppressing the adaptive immune responses (3). Recent studies have shown that COVID-19 patients, compared to healthy controls, had significantly lower absolute numbers of total lymphocytes and subsets of CD3+, CD4+, and CD8+ T cells, CD19+ B cells, and CD56+ NK cells (4).

Concerning this, lymphocytes’ survival is known to be closely regulated by autophagy (5). Autophagy is a metabolic process involved in the degradation of intracellular components *via* lysosomal machinery, by engulfment of damaged proteins and organelles in double-membrane vesicles called autophagosomes (6). Furthermore, the crosstalk between autophagy and apoptosis, crucial for cell survival, has been previously shown (7), and several studies correlated the disruption of autophagy with the contemporary increase of apoptotic cell death in several diseases (8).

In COVID-19 patients, the accumulation of autophagosomes promoted by SARS-CoV-2 infection may exacerbate the processes of apoptotic cell death (9), and higher levels of apoptosis in lymphocytes could be related to lymphopenia detected in severe COVID-19 conditions (10).

To date, the mechanism underlying the kinetics of peripheral lymphocyte changes due to COVID-19 is unclear.

This study sought to investigate the relationship between COVID-19 and the autophagy of circulating peripheral blood mononuclear cells (PBMCs) to better characterize lymphopenia during SARS-CoV-2 infection.

## Material and Methods

### Patients

Eighteen COVID-19 patients attending the University Hospital Policlinico Umberto I, Sapienza University of Rome, were enrolled. The study was approved by the local ethics committee (protocol number 0586/20), and informed consent was obtained from each patient. As a control group, twelve age- and sex-matched healthy donors (HDs) were studied. From every participant in the study, a blood sample was collected to purify PBMCs by Ficoll-Hypaque. Sera were obtained by centrifugation at 3,500 rpm for 15 min and then stored at −20°C until use for *in vitro* treatments.

### Cell Cultures and Treatments

After counting, isolated PBMCs were cultured on 6-well dishes at a concentration of 2 × 10^6^ PBMCs/ml/well in RPMI-1640 medium supplemented with 10% fetal bovine serum (FBS), 2 mM of glutamine, and 50 μg/ml of gentamycin and treated with the following: i) lysosomal inhibitors E64d and pepstatin A (both at 10 μg/ml) for 2 h before the end of culture; ii) 10% COVID-19 or HD sera (as a replacement of FBS) for 24 h, selected by preliminary time-course experiments (11).

### Autophagy Marker Detection by Western Blotting

On the same day of the blood sample collection and after *in vitro* treatment with sera, PBMCs were lysed in radioimmunoprecipitation assay (RIPA) buffer (100 mM of Tris-HCl pH 8, 150 mM of NaCl, 1% Triton X-100, 1 mM of MgCl, 25 mM of NaVO_4_, and protease-inhibitor mixture). Lysates were loaded onto a 15% sodium dodecyl sulfate–polyacrylamide gel electrophoresis (SDS-PAGE) in denaturing conditions. Subsequently, Western blotting was performed, and the membranes were incubated with a rabbit anti-human LC3IIB and a rabbit anti-p62 Abs (1:1,000 diluted in Tris-buffered saline containing milk at 5%) (Cell Signaling, Danvers, MA, USA) (12). Peroxidase-conjugated goat anti-rabbit IgG was used as a secondary Abs, and the reaction was developed using the SuperSignal West Pico Chemiluminescent Substrate (Millipore, Billerica, MA, USA). A rabbit anti-human β-actin Ab was used to ensure the presence of equal amounts of protein. Quantification of protein expression was performed by densitometry analysis.

### Flow Cytometry for Apoptosis and Autophagy Analysis

As before, PBMC apoptosis was analyzed using a fluorescein isothiocyanate (FITC)-conjugated annexin V (AV) and phycoerythrin (PE)-conjugated propidium iodide (PI) apoptosis detection kit, according to the manufacturer’s protocol (MBL, Woburn, MA, USA). In particular, 1 × 10^6^ PBMCs were stained in AV buffer with AV-FITC and PI-PE (1:100 diluted) for 10 min in the dark. After washing, cells were transferred into fluorescence-activated cell sorting (FACS) tubes and analyzed. The acquisition was performed on a FACSCalibur cytometer (BD, San Jose, CA, USA), and 10,000 events/sample were run. Data were analyzed using the Cell Quest Pro software (11).

Autophagy levels in CD4+, CD8+, CD19+ lymphocytes, and CD14+ cells from COVID-19 were also detected using the Cyto-ID autophagy detection kit (Enzo Life Sciences, Farmingdale, NY, USA). The probe used in this kit consists of cationic amphiphilic tracer dye that stains autophagolysosomes (13). For the immunophenotyping analysis, see the **Supplementary Material**.

### Immunofluorescence of Autophagy Markers

An indirect immunofluorescence assay was developed on freshly isolated PBMCs from both COVID-19 patients and HDs, prepared by the cytospin technique. In detail, after slide preparation, a cell suspension of 0.5 × 10^6^ cells/ml was gently pipetted into the cytofunnel and centrifuged at 1,000 × *g* for 5 min. The removed slides, after drying at least 2 h at room temperature (RT), were fixed with paraformaldehyde (PFA) at 4% in phosphate-buffered saline (PBS) for 15 min at RT, then treated with PBS-Triton X-100 0.1% for 10 min, and blocked with 3% bovine serum albumin (BSA) in PBS for 30 min at RT. Successively, slides were incubated overnight at 4°C with the following primary antibodies: rabbit anti-human LC3IIB (Cell Signaling; 1:100 diluted) and a mouse anti-human lysosomal-associated membrane protein 1 (LAMP1) (Invitrogen, Carlsbad, CA, USA; 1:100 diluted). The day after, slides were washed three times in PBS, and Tetramethylrhodamine-isothiocyanate (TRITC)–anti-rabbit and FITC–anti-mouse IgG (Sigma Aldrich) were added and incubated for 45 min at RT. After incubation, slides were washed three times in PBS, and the second wash was performed with the addition of Hoechst (Molecular Probes, Eugene, OR, USA) for nuclear staining. Fluorescence was analyzed by a fluorescence microscope (Olympus, Tokyo, Japan; BX52). Image acquisition and processing were conducted by IAS 2000 software. Morphometric analysis of cellular expression was carried out by counting at least 200 cells in different microscopic fields at magnifications of 50× and 100×.

### Statistical Analysis

Data are expressed as means ± SD. Results were analyzed with GraphPad Prism 6. The Mann–Whitney test or Student’s t-test was used to compare quantitative variables in different groups, and the chi-square test was used to test a correlation between categorical variables. Spearman’s rank correlation coefficient was applied for the calculation of the correlation between parallel variables in single samples. Values of *p* < 0.05 were considered statistically significant.

## Results

### Serological Characteristics of COVID-19 Patients

The serological characteristics of COVID-19 patients are summarized in **Table 1**.

**Table 1:** Summarizes serological features of the enrolled patients.

PATIENT	GENDER	HAEMOGLOBIN (g/dL)	PLATELET (10^3^X µL)	LEUKOCYTE (10^3^X µL)	NEUTROPHILS (10^3^X µL)	*LYMPHOCYTES (10^3^X µL)*	EOSINOPHILS (10^3^X µL)	ESR (mm/h)	CPR (µg/L)	Ferritin (ng/mL)	D-dimers	IL-6 (pg/mL)
											(ng/mL)	
**HIGH INFLAMMATORY PROFILE (HIP)**												
**3**	F	14.6	228	5860	4260	990	40	NA	3700	96	525	7.75
**5**	M	13.3	316	10590	9070	750	10	61	61400	667	344	49.62
**8**	F	9.9	112	4580	3550	660	10	2	6600	72	170	14.06
**9**	M	8.4	237	2310	1930	220	10	56	139800	614	730	NA
**12**	M	10	98	5810	4860	370	200	NA	NA	NA	NA	NA
**16**	M	12.1	483	12890	11580	790	120	NA	1390	NA	1079	NA
**LOW INFLAMMATORY PROFILE (LIP)**												
**6**	M	12.4	222	7410	4990	1830	30	22	10300	150	1775	21.43
**7**	M	14.3	237	4640	2610	1610	20	38	10600	230	658	14.83
**11**	M	16.1	130	14240	9270	1800	10	NA	97800	NA	NA	NA
**15**	M	11.6	387	10160	5930	3350	60	NA	12900	NA	455	NA
**10**	F	14.6	264	11800	9680	1113	400	NA	3640	NA	1194	NA
**14**	M	13.7	365	8030	6300	1150	20	NA	5080	NA	1574	NA
**1**	M	15	282	8380	5866	1187	243	9	2900	147	170	4.65
**2**	M	12.5	244	7700	4970	1960	160	NA	350	22	467	NA
**4**	M	15.1	173	7040	3840	2270	150	NA	1300	247	170	3.08
**13**	F	12.9	314	5720	3830	1160	20	NA	4520	NA	NA	NA
**17**	M	8.9	240	74160	3930	64300	100	NA	3100	NA	NA	NA
**18**	F	NA	NA	NA	NA	NA	NA	NA	NA	NA	NA	NA

Laboratory abnormalities findings are reported in red. Patients were divided into two groups. The first group, High Inflammatory Profile (HIP), includes patients who reported lymphopenia (Lymphocyte <1000 cells/μL), and almost one of the following serological features: Platelets <100.000/μL, IL-6 >5.9 pg/mL, CRP >5000 µg/L, D-dimers >1000 μg/L , Ferritin >250 ng/mL, ESR > 30 mmh. The second group didn’t meet the above mentioned criteria and was defined Low Inflammatory Profile (LIP). Green lines displayed patients who showed high PBMC LC3IIB level (>2.5) based on the Western blot analysis. Not Available (NA).

Patients were divided into two groups, high inflammatory profile (HIP) and low inflammatory profile (LIP), according to serological and laboratory features.

Specifically, HIP patients displayed lymphocyte < 1,000 × 10^9^/L with the addition of one of the following serological features: platelets < 100,000 × 10^9^/L, IL-6 > 5.9 pg/ml, C-reactive protein (CRP) > 5,000 mg/ml, D-dimers > 1,000 ng/ml, ferritin > 250 ng/ml, and erythrocyte sedimentation rate (ESR) > 30 mm/h.

LIP patients did not meet the abovementioned criteria.

### Spontaneous Autophagy and Apoptosis in COVID-19 Patients and Healthy Donors

**Figure 1:**
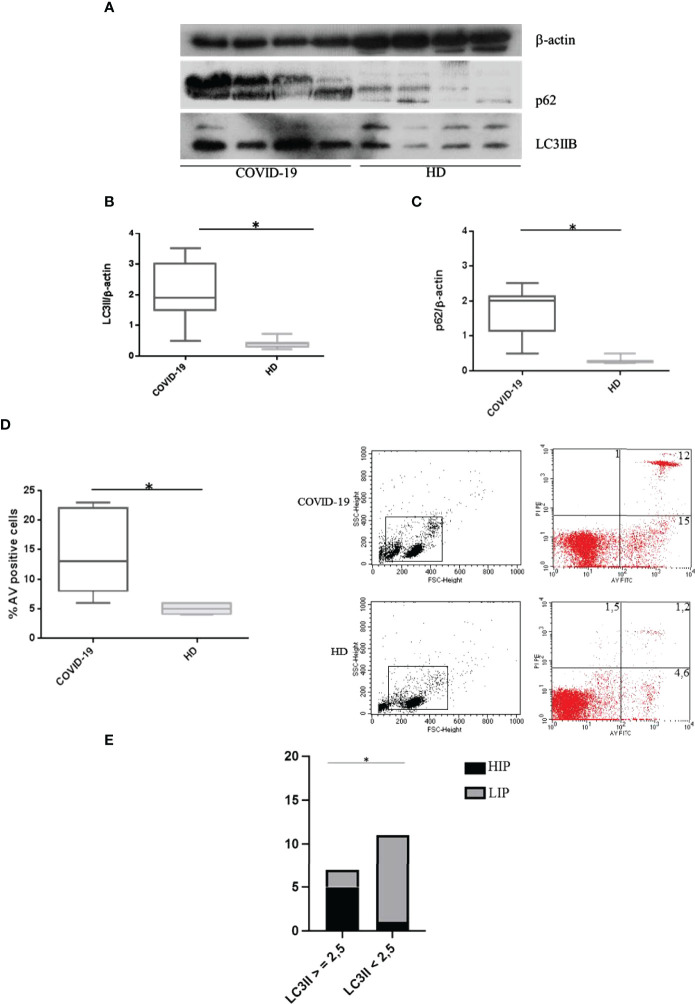
Spontaneous autophagy and apoptosis in COVID-19 patients and HDs. **(A)** LC3IIB and p62 Western blotting analysis of PBMC lysates (30 μg/lane) from 4 COVID-19 patients and 4 HDs. Blots shown are representative of independent experiments performed on COVID-19 patients (*n* = 18) and HDs (*n* = 12). Quantification of LC3IIB **(B)** and p62 **(C)** levels relative to β-actin is also shown (mean with range is presented) (Mann–Whitney test). **(D)** Flow cytometry analysis of PBMC apoptosis. Data are referred to as AV-positive cells and are presented as independent experiments performed in PBMC from COVID-19 patients (*n* = 18) and HDs (*n* = 12) (Mann–Whitney test). **(E)** Contingency: prospective data (chi-square test) between LC3IIB level and HIP/LIP patients. Values are expressed as means ± SD. *p < 0.05. COVID-19, coronavirus disease 2019; HDs, healthy donors; PBMC, peripheral blood mononuclear cell; AV, annexin V; HIP, high inflammatory profile; LIP, low inflammatory profile.

Basal autophagy and apoptosis in PBMCs from COVID-19 patients and HDs were evaluated, and our results showed significantly higher LC3IIB levels in COVID-19 patients compared to HDs (*p* < 0.0001) (**Figures 1A, B**), presuming an upregulation of autophagy. In addition, p62 levels were significantly higher in COVID-19 patients than in HDs (*p* < 0.0001) (**Figures 1A, C**), underlining an accumulation of p62 due to an autophagy impairment in these patients. Furthermore, freshly isolated PBMC apoptosis was higher in COVID-19 patients in comparison to HDs (*p* = 0.0186) (**Figure 1D**).

Interestingly, we observed that patients showing high levels of LC3IIB (>2.5) were classified as HIP (**Figure 1E**).

Moreover, COVID-19 immunophenotyping studies revealed higher levels of autophagolysosome formation in CD4+ and CD8+ cells compared to CD14+ cells (*p* = 0.04 and *p* = 0.0007, respectively); however, the autophagy level in CD19+ cells was not higher as compared to CD14+ cells (**Figure 1A**—**Supplementary Material**).

### Autophagy Block in Peripheral Blood Mononuclear Cells From COVID-19 Patients and Its Correlation With Apoptosis

**Figure 2:**
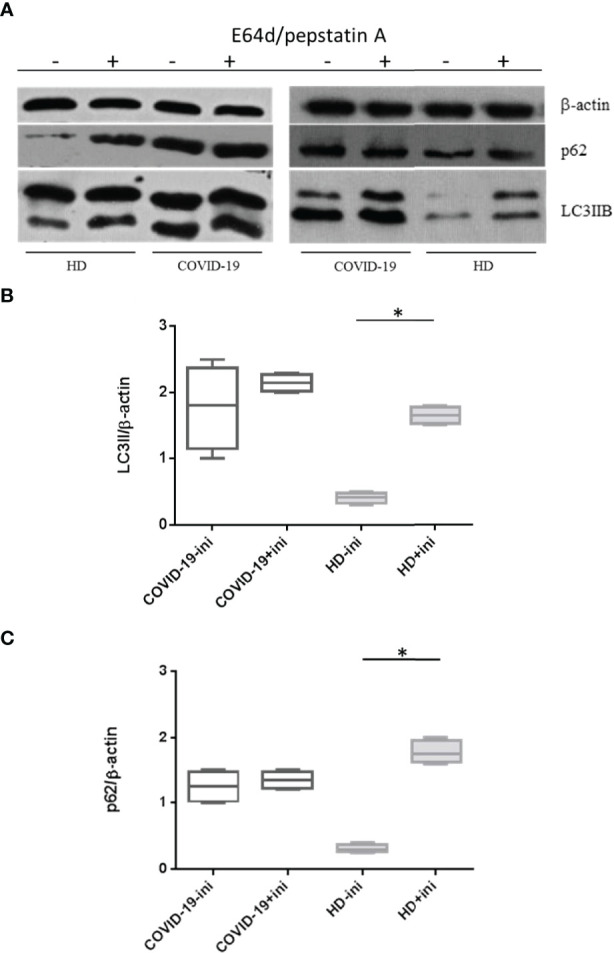
Effects of lysosomal inhibitors E64d and pepstatin A on PBMC autophagy. **(A)** LC3IIB and p62 Western blotting analysis of PBMC lysate (30 μg/lane) from 2 representative COVID-19 patients and 2 HDs (10 independent experiments). Where indicated, cells were treated with the lysosomal inhibitors E64d and pepstatin A indicated as “ini.” Densitometry analysis of LC3IIB **(B)** and p62 **(C)** levels relative to β-actin is also shown (Mann–Whitney test). Values are expressed as means ± SD. *p < 0.05. PBMC, peripheral blood mononuclear cell; COVID-19, coronavirus disease 2019.

According to these results, we hypothesized an autophagy dysfunction and/or blockade in PBMCs from COVID-19 patients. Thus, we assessed *in vitro* experiments with lysosomal inhibitors E64d and pepstatin A. In PBMCs from patients affected by COVID-19, LC3IIB levels did not change in the presence of lysosomal inhibitors, compared to untreated cells (*p* > 0.05) (**Figures 2A, B**). As previously demonstrated (11), we observed an increase in LC3IIB levels in HD PBMCs treated with lysosomal inhibitors *versus* untreated (*p* = 0.0286) (**Figures 2A, B**). Concurrently, p62 levels did not change in COVID-19 PBMCs treated with lysosomal inhibitors but increased in treated PBMCs from HDs, compared to untreated in both conditions (*p* > 0.05 and *p* = 0.0286, respectively) (**Figures 2A, C**). This result explains how a blocked mechanism does not undergo modifications under further inhibitory conditions. So PBMCs from COVID-19 patients, in which the mechanism is blocked, contrary to HD PBMCs, do not respond to inhibitors, and neither LC3IIB nor p62 nor apoptosis changed under E64d/pepstatin A treatment.

**Figure 3:**
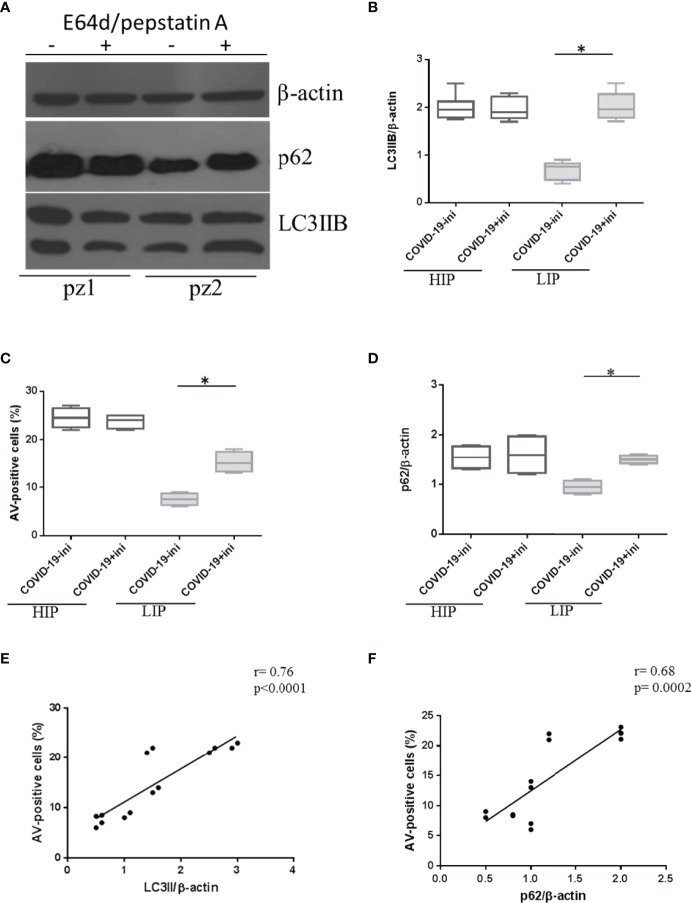
Expression level of autophagy and apoptosis markers in HIP and LIP COVID-19 patients treated with lysosomal inhibitors. **(A)** LC3IIB and p62 Western blotting analysis of PBMC lysate (30 μg/lane) from 2 representative COVID-19 patients (1 HIP indicated as pz1 and 1 LIP indicated as pz2 of the 18 analyzed). Densitometry analysis of LC3IIB **(B)** and p62 **(D)** levels relative to β-actin is also shown (Mann–Whitney test). **(C)** Flow cytometry analysis of PBMC apoptosis. Data are referred to as AV-positive cells and are presented as independent experiments performed in PBMCs from COVID-19 patients (*n* = 18) (Student’s t-test). Where indicated, cells were treated with the lysosomal inhibitors E64d and pepstatin A indicated as “ini.” Values are expressed as means ± SD. *p < 0.05. **(E, F)** Correlation and linear regression analysis of apoptosis and LC3IIB and p62 levels in PBMCs from COVID-19 patients. HIP, high inflammatory profile; LIP, low inflammatory profile; PBMC, peripheral blood mononuclear cell; COVID-19, coronavirus disease 2019; AV, annexin V.

However, we observed variability in p62 expression levels among patients in the COVID-19 cohort (**Figure 3**). In detail, LIP patients were susceptible to E64d/pepstatin A treatment (*p* < 0.0001 for autophagy, *p* = 0.002 for apoptosis, *versus* untreated) (i.e., pz 2 in Western blotting of **Figures 3A–C**), while HIP patients did not respond to autophagy inhibitors (*p* > 0.05) [i.e., pz 1 in (**Figure 3A–C**)].

As expected, in HIP patients displaying a basal block of autophagy and higher levels of apoptosis, the amount of p62 in the presence of lysosomal inhibitors *versus* untreated did not change (*p* > 0.05) (**Figure 3D**); on the contrary, LIP patients presenting basal levels of autophagy and apoptosis, similar to HDs, showed an accumulation of p62 under lysosomal inhibitors treatment (*p* = 0.0008) (**Figure 3D**).

In addition, a positive correlation between autophagy markers and apoptosis was observed in PBMCs from COVID-19 patients (**Figures 3E, F**), leading us to speculate that autophagy hijacking in PBMCs from COVID-19 patients is directly involved in cell death.

**Figure 4:**
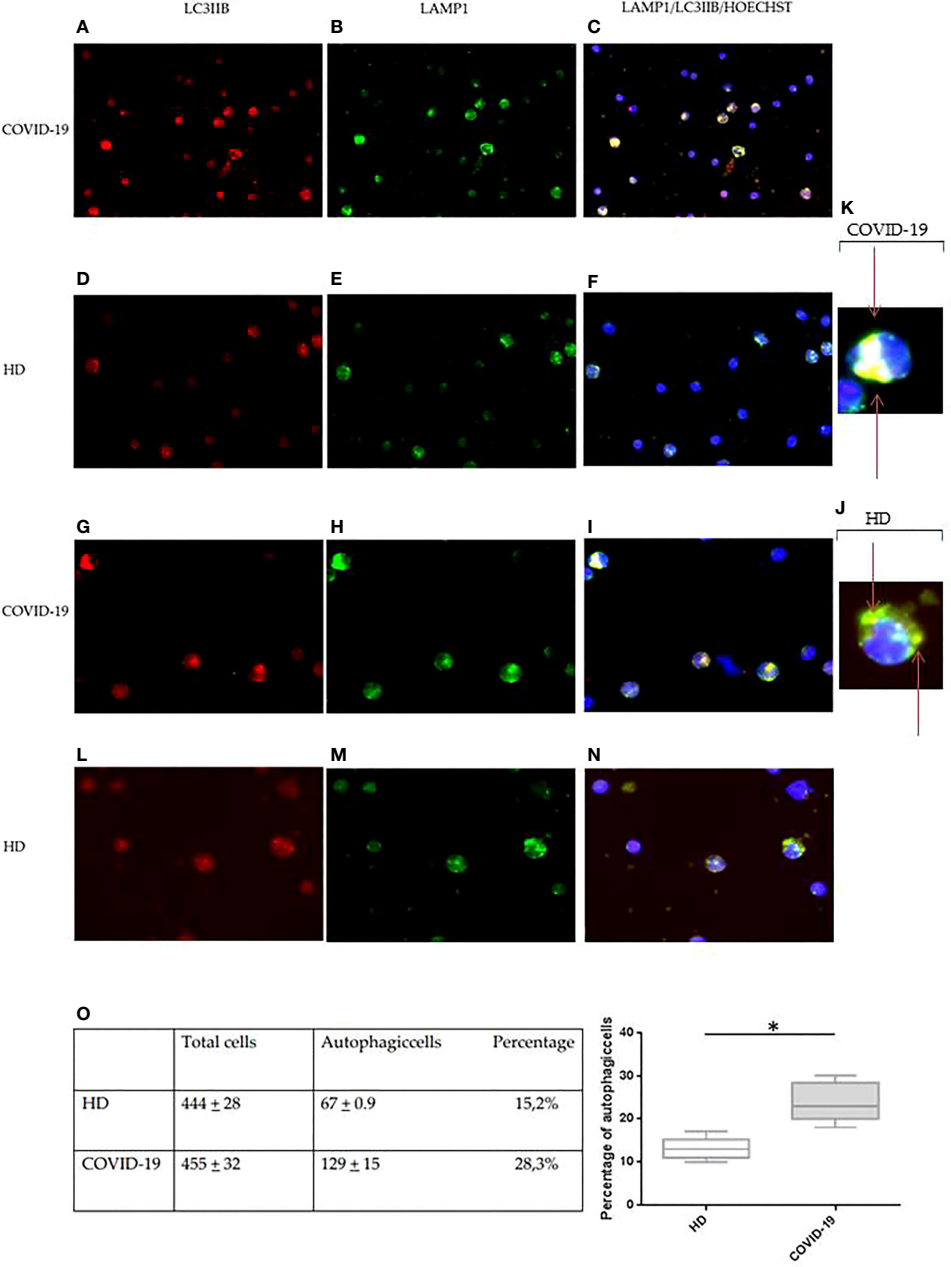
Immunofluorescence analysis of autophagy in PBMCs from COVID-19 patients and HDs. LC3IIB (red fluorescence) and LAMP1 (green fluorescence) expression in PBMCs from COVID-19 patients **(A, B, G, H)** and HDs **(D, E, L, M)**. Intracellular autophagolysosome formation detection by colocalization between LC3IIB and LAMP1 (yellow fluorescence) in PBMCs from COVID-19 patients **(C, I)** and HDs **(F, N)**. **(A–F)** Magnification, 50×. **(G-I, L-N)** Magnification, 100×. **(J, K)** Magnification, x200. **(O)** Table of total cells and autophagic cells percentage and analysis of the percentage of autophagic cells in COVID-19 and HD PBMCs (Student’s t-test). Values are expressed as means ± SD. *p < 0.05. PBMC, peripheral blood mononuclear cell; COVID-19, coronavirus disease 2019; HDs, healthy donors.

To confirm the alteration in the autophagy process, immunofluorescence analysis was also used to verify the expression levels of the autophagosome marker LC3IIB and the lysosome marker LAMP1. Specifically, the presence of intracellular autophagolysosomes indicating the ongoing PBMC autophagy was assessed by the detection of colocalization between LC3IIB and LAMP1, revealed by the yellow color in immunofluorescence analysis (**Figure 4**).

In PBMCs from COVID-19 and HDs, the expression of LC3IIB and LAMP1 was diffusely detectable (**Figure 4**). However, the expression of both LC3IIB puncta and LAMP1 was more regularly distributed in PBMCs from HDs (**Figures 4D, E, L, M**) as compared to PBMCs from COVID-19 (**Figures 4A, B, G, H**).

Additionally, the aberrant autophagolysosome formation was commonly observed in PBMCs from COVID-19 patients (**Figures 4C, I**), showing a diffuse pattern, while autophagolysosomes of PBMCs from HDs had the typical dotted pattern (**Figures 4F, N**). This result confirmed an alteration of the degradation cellular mechanism.

The autophagic cells and the total stained cells were then counted in 10 different selected fields per specimen, and autophagic cells resulted from 28.1% in COVID-19 PBMCs and 15.2% in HD PBMCs (*p* = 0.0001) (**Figure 4O**).

### Sera From COVID-19 Patients Differentially Modulate Autophagy and Apoptosis *In Vitro*

Our findings suggest the presence of soluble factors able to hijack PBMCs autophagy and apoptosis in COVID-19 patients’ sera. To confirm this, we conducted *in vitro* studies treating PBMCs from HDs with COVID-19 patients’ sera for 24 h. For this set of experiments, we chose sera from a pool of COVID-19 patients classified as HIP or LIP.

**Figure 5:**
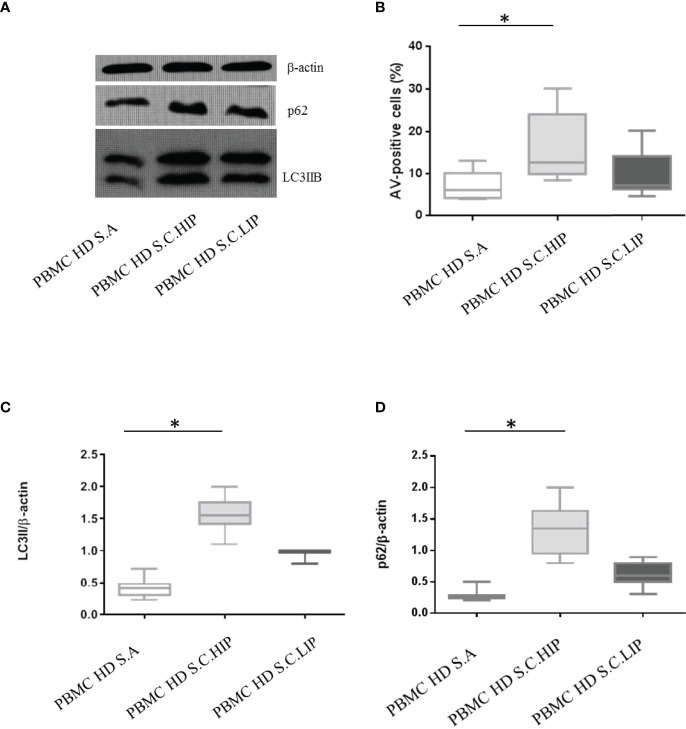
Effects of sera from patients with COVID-19 on HD PBMC autophagy and apoptosis. **(A)** LC3IIB and p62 Western blotting analysis of PBMC lysate (30 μg/lane) from 1 representative HDs *in vitro* treated with autologous sera (S.A), with sera from 1 HIP COVID-19 patients (S.C.HIP) and from 1 COVID-19 classified as LIP (S.C.LIP) **(B)** Flow cytometry analysis of PBMC apoptosis. Data are referred to as AV-positive cells and are presented as 10 independent experiments performed on HDs *in vitro* treated with sera from HIP COVID-19 patients and COVID-19 classified as LIP (Student’s t-test). Densitometry analysis of LC3IIB **(C)** and p62 **(D)** levels relative to β-actin is also shown (10 independent experiments) (Mann–Whitney test). Values are expressed as means ± SD. *p < 0.05. PBMC, peripheral blood mononuclear cell; HD, healthy donor; COVID-19, coronavirus disease 2019; HIP, high inflammatory profile; LIP, low inflammatory profile.

Interestingly, sera from COVID-19 patients were able to modulate autophagy and apoptosis based on their inflammatory profile (**Figure 5**). PBMCs from HDs *in vitro* cultured with sera from COVID-19 patients classified as HIP, displayed high levels of apoptosis and a block of autophagy, as confirmed by LC3IIB and p62 accumulation (*p* = 0.02, *p* = 0.002, and *p* = 0.0007 *versus* untreated, for apoptosis, LC3IIB, and p62 levels, respectively). In contrast, sera from patients classified as LIP were not able to modulate apoptosis and autophagy in PBMCs from HDs *in vitro* (*p* > 0.05 for all parameters) (**Figures 5A–D**).

### Correlation Between Autophagy Block and Lymphopenia

**Figure 6:**
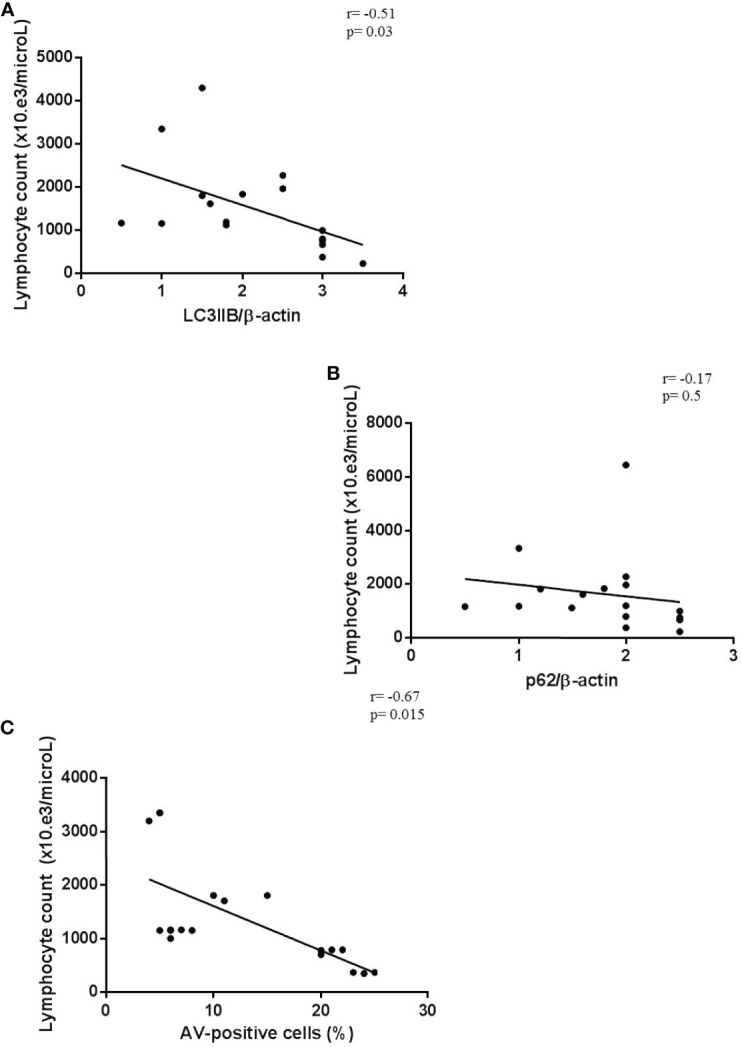
Correlation between lymphopenia and autophagy/apoptotic markers. **(A–C)** Correlation and linear regression analysis of lymphocyte count and LC3IIB and p62 levels, and apoptosis in PBMCs from COVID-19 patients (*n* = 18) (Spearman’s rank correlation). PBMC, peripheral blood mononuclear cell; COVID-19, coronavirus disease 2019.

Finally, we observed a significant correlation between lymphopenia and autophagic/apoptotic markers in COVID-19 PBMCs. In particular, peripheral lymphocyte count negatively correlated with LC3IIB and p62 levels and with cell apoptosis rate (**Figures 6A–C**).

These results suggest that in these patients the inflammatory condition could interfere with the major mechanism of cellular survival, which is autophagy, leading to lymphocyte death by apoptosis.

## Discussion

In our study, we firstly showed a block of autophagy in PBMCs from COVID-19 patients who displayed high expression of LC3IIB and p62 levels. These results were strengthened by experiments under lysosomal inhibition conditions, in which lysosomal proteases did not affect autophagy levels in HIP COVID-19 PBMCs but affected autophagy only in those patients showing a LIP.

It is well known that autophagy is a mechanism involved in the most important steps of the immune response, such as intracellular pathogen sensing, lymphocyte development, homeostasis, and survival (14). Although autophagy is a cell survival mechanism, it is also linked to cell death, through the interaction with apoptosis-related proteins (15).

In the present study, patients with a HIP showed a block of autophagy and a concomitant high percentage of PBMCs undergoing apoptosis. This could mean that cellular death is the direct consequence of the aberration of the survival mechanism. This hypothesis is supported by the increased apoptosis and the block of autophagy in PBMCs from HDs *in vitro* treated with COVID-19 sera showing HIP.

In PBMCs from COVID-19 patients, at a molecular level, the interplay between lymphocyte autophagy and apoptosis has never been investigated, but our results indicate cytokines or circulating proinflammatory molecules as a possible main culprit. In literature, it is known that many circulating cytokines, such as IL-6, TNF-alpha, and BlyS, as in autoimmune/inflammatory diseases, could be involved in the regulation of this process (16, 17), causing a cytokine storm that could contribute to a more severe COVID-19 disease also by exhaustion of lymphocytes (18). Data from the present study suppose a possible effect of the virus on lymphocyte autophagy dysregulation, likely as a result of a cytokine storm (19).

To strengthen our hypothesis on the autophagy blockade in circulating lymphocytes from COVID-19 patients, the analysis of the autophagolysosome formation level in the PBMC subpopulations, which is significantly higher in lymphocytes than in monocytes, contributes to lymphocyte death. In this regard, we observed also a strong positive correlation between autophagy markers and apoptosis.

In addition, the indirect correlation between autophagy/apoptotic markers and lymphocyte count demonstrates the interaction between autophagy block and the concomitant apoptosis increase with the decrease in circulating lymphocytes. Moreover, lymphocytes are crucial in the maintenance of immune homeostasis and inflammatory response; thus, the understanding of the mechanism of reduced blood lymphocyte levels could provide an additional strategy for the treatment of COVID-19 (20).

In our previous study, we speculated the direct infection of the virus on lymphocytes, resulting in death due to lymphocyte expression of the coronavirus receptor ACE2 (19). In accordance with our hypothesis, Tan et al. added the direct destruction of lymphatic organs by the virus as a possible cause of lymphocyte decline, with the inflammatory cytokine storm that leads to lymphocyte apoptosis (20). The authors concluded that lymphopenia is an indicator of the severity of COVID-19 hospitalized patients and suggested including the evaluation of blood lymphocyte percentage in the guidelines for the diagnosis of COVID-19 (21). Our results reinforce this suggestion and add new knowledge on the mechanisms underlying lymphopenia in COVID-19 patients.

Considering these results, drugs targeting autophagy could represent an important issue, worthy to be considered as a new therapeutic strategy in the context of COVID-19. Although additional studies are needed to confirm our hypotheses, since autophagy and apoptosis are usually involved in many disease conditions, this study provides intriguing data to better understand the mechanisms underlying COVID-19 and causing the disease progression.

The strength of this study is the use of different experimental approaches to confirm a block of autophagy in PBMCs from COVID-19 patients. Despite the promising information obtained from the analysis of COVID-19 PBMC homeostasis, limitations are present. The number of patients is small due to the difficult enrollment during the pandemic period. In addition, we do not know the patients’ therapy that could interfere with the investigated mechanisms.

## Data Availability Statement

The raw data supporting the conclusions of this article will be made available by the authors, without undue reservation.

## Ethics Statement

The study was approved by the ethics committee of Sapienza University of Rome (protocol number 0586/20). The patients/participants provided their written informed consent to participate in this study.

## Author Contributions

All authors have made a substantial, direct, and intellectual contribution to the work and approved the final manuscript.

## Conflict of Interest

The authors declare that the research was conducted in the absence of any commercial or financial relationships that could be construed as a potential conflict of interest.

## Publisher’s Note

All claims expressed in this article are solely those of the authors and do not necessarily represent those of their affiliated organizations, or those of the publisher, the editors and the reviewers. Any product that may be evaluated in this article, or claim that may be made by its manufacturer, is not guaranteed or endorsed by the publisher.

## Abbreviations

COVID-19, coronavirus disease 2019; FBS, fetal bovine serum; HDs, healthy donors; HIP, high inflammatory profile; LIP, low inflammatory profile; PBMCs, peripheral blood mononuclear cells; SARS-CoV-2, severe acute respiratory syndrome coronavirus 2
