# Supplementary material for: Autophagy Hijacking in PBMC From COVID-19 Patients Results in Lymphopenia
Source: Front Immunol. 2022 May 30;13:903498. doi: 10.3389/fimmu.2022.903498 (PMC9196331; doi:10.3389/fimmu.2022.903498)
Supplement: Supplementary file 1 [file DataSheet_1.pdf]

Supplementary material

Autophagy hijacking in PBMC from COVID-19 patients results in lymphopenia

Barbati C. and Celia A.I. et al

Flow cytometry for autophagy analysis in PBMC's subpopulations from COVID-19

Autophagy levels in PBMC's subpopulations from COVID-19 was assessed by evaluating the autophagic flux (the active fusion between autophagosomes and lysosomes) using a Cyto-ID kit (Enzo Life Sciences), which allows for the identification and quantification of autophagosome–lysosome fusion. This kit measures autophagic vacuoles and monitors autophagic flux in lysosomally inhibited live cells using a dye that selectively labels accumulated autophagic vacuoles. Briefly, PBMCs (1×106 cells for each COVID-19 patient) were stained with anti-CD4, anti-CD8, anti CD-19, anti-CD14 antibodies and control mouse IgG (Miltenyi Biotec). After 20 minutes on ice in the dark, cells were washed in PBS and then resuspended in DMEM without phenol red, supplemented with 5% FBS and incubated with CYTO-ID® Green Detection Reagent for 30 minutes in the dark, at 37 °C. 100,000 events per sample were run on FACSCalibur flow cytometer (BD Biosciences) and data were analyzed using the Cell Quest Pro software (BD Biosciences). Values were shown as mean fluorescence intensities (MFI) of Cyto-ID.

Figure legend supplementary material

Figure 1. supplementary material

Figure 1. Flow cytometer analysis of autophagy in circulating lymphocytes and monocytes in COVID-19 patients. (**A**) Autophagy levels in circulating CD4+, CD8+, CD19+ and and CD14+ cells from COVID-19 patients. Red dot plot of CD4+,CD8+,CD19+ and CD14+ are gated from R1. Isotype control staining is represented by the full purple histogram and Cyto-ID-labeled lymphocytes by the green-solid line. Histograms are sequentially gated from R2,R3,R4 and R5, respectively. Data are presented as box and whisker plots and values shown as mean fluorescence intensities (MFI) of Cyto-ID. Values are expressed as means ± sd. *$P < 0.05$. (Mann–Whitney test).
