# Supplementary figures and images for: Autophagy Hijacking in PBMC From COVID-19 Patients Results in Lymphopenia
Source: Front Immunol. 2022 May 30;13:903498. doi: 10.3389/fimmu.2022.903498 (PMC9196331; doi:10.3389/fimmu.2022.903498)

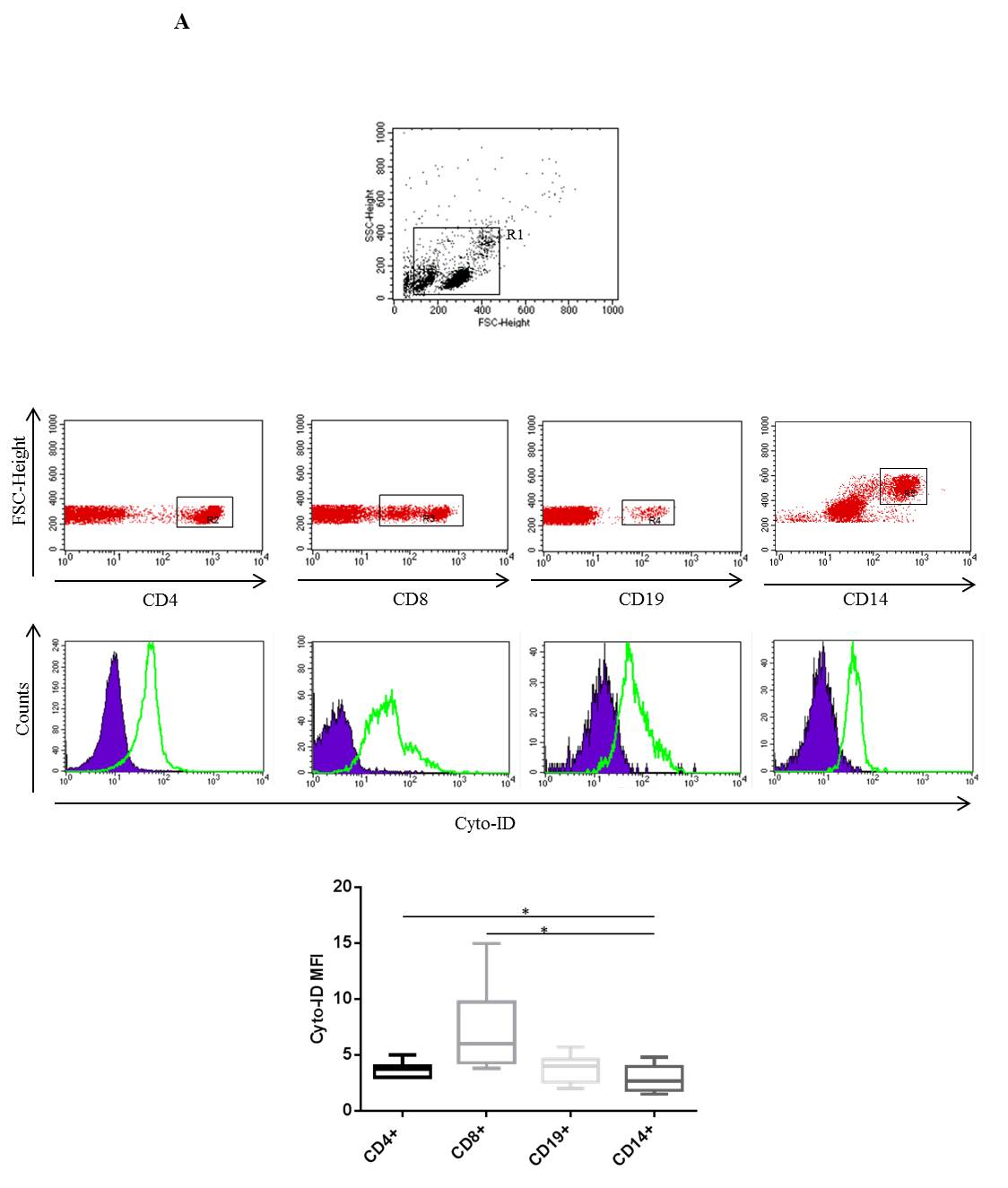

Supplement: Supplementary file 2 [file Image_1.jpeg]
